# Violence against healthcare workers during the COVID-19 pandemic: a cross-sectional survey at Cairo University Hospital

**DOI:** 10.3389/fpubh.2023.1277056

**Published:** 2023-11-16

**Authors:** Salma Abdelrehim Seddik, Rehab Abdelhai, Ahmed Taha Aboushady, Ahmed Essam Nawwar, Rania Assem El Essawy, Amira Aly Hegazy

**Affiliations:** ^1^Department of Public Health and Community Medicine, Faculty of Medicine, Cairo University, Cairo, Egypt; ^2^Faculty of Medicine, Alexandria University, Alexandria, Egypt; ^3^Department of Ophthalmology, Faculty of Medicine, Cairo University, Cairo, Egypt

**Keywords:** violence, healthcare worker, COVID-19, health service research, mental health

## Abstract

**Introduction:**

Healthcare workers have a significant chance of experiencing violence, with physical violence impacting anywhere from 8 to 38% of healthcare professionals throughout their careers. Besides physical abuse, many healthcare workers are subject to verbal aggression or threats, with patients and visitors being the most frequent sources of such incidents.

**Methods:**

This research examines the work atmosphere of healthcare professionals at Kasr Al-Aini University Hospital in Cairo, Egypt, during the pandemic. The study aims to evaluate the frequency of violence toward healthcare workers and health professionals training through a cross-sectional survey conducted among them. The research was conducted on Egyptian healthcare workers over 6 months, from November 2020 until the end of January 2021, using convenience sampling in a cross-sectional study. Over half of the respondents reported experiencing violence.

**Results:**

Among those who experienced violence, 93% reported verbal aggression, 43% reported physical and verbal abuse, and 59% claimed that violence increased during the pandemic. Additionally, 97% of those who experienced violence reported it occurring within the 4 months following the survey. About 42.5% of the respondents were female, and nearly 65% were over 30. 82% of the respondents did not receive training on handling violence while performing their job.

**Conclusion:**

This study highlights the high prevalence of verbal assaults in healthcare settings, primarily by patients’ families or acquaintances. Despite reporting such incidents, most respondents did not see any significant government action. Furthermore, the COVID-19 pandemic did not significantly change the frequency of violent incidents, indicating that the root causes of violence are systemic and extend beyond the pandemic. These findings underscore the need for systemic changes in healthcare organizations to address and prevent violence against healthcare workers.

## 1 Introduction

Lessons learned from past outbreaks of infectious diseases, such as severe acute respiratory syndrome (SARS) in 2003, Middle East respiratory syndrome (MERS) from 2013 to 2016, and Ebola from 2014 to 2016, indicate that healthcare professionals face significant challenges during such crises (1, 2). While each outbreak has its unique characteristics, they all share common elements, including heavy workloads, changes in tasks and responsibilities, increased risk of infection, more demanding working conditions due to protective measures, and exposure to emotionally distressing events and trauma (2). Studies conducted after the SARS, MERS, and Ebola outbreaks have shown that persistent exposure to stress, anxiety, trauma, violence, sleep deprivation, and fatigue in the short term can lead to errors and decreased work performance among healthcare professionals (1, 2). Long-term effects may include burnout, depression, anxiety, and post-traumatic stress disorder. Various social and occupational factors impact the mental well-being of healthcare professionals, underscoring the importance of early intervention to minimize harm (1, 2).

Moreover, healthcare professionals worldwide face a high risk of violence, with physical violence affecting between 8 and 38% of healthcare workers during their careers. In addition to physical violence, many healthcare workers are threatened or subjected to verbal aggression, with patients and visitors being the most common perpetrators (3).

Violence against healthcare workers is unacceptable and concerning, negatively impacting healthcare staff’s psychological and physical well-being. Moreover, this type of violence can reduce healthcare workers’ job motivation, ultimately compromising the quality of care and putting healthcare provision at risk. Additionally, it can result in significant financial losses for the healthcare sector. Several studies have demonstrated the different types of consequences of workplace violence in healthcare settings (4–6).

The coronavirus disease 2019 (COVID-19) has garnered significant global attention since its first identification in China in December 2019 (7). Egypt announced its first confirmed COVID-19 case on February 14, 2020, and has since implemented measures such as evacuating Egyptian citizens from heavily infected countries, closing schools and universities, and enforcing home-based work for civil servants (8, 9). A confinement law banning all public meetings and gatherings was also implemented on March 24, 2020 (10).

Approximately half of the general population, in China as reported by Wang et al. (11), rated the psychological impact of the COVID-19 outbreak as either moderate or severe. However, HCWs have been identified by the World Health Organization as a particularly vulnerable group at risk of experiencing a wide range of mental and physical health issues due to their direct or indirect involvement in caring for COVID-19 patients (12). HCWs face an increased threat of transmission (13), due to their frontline role in treating patients with high viral loads and inadequate personal protective equipment (14). Moreover, HCWs are exposed to severe stress, emotional burdens, long working hours, worries about contracting the virus or infecting their loved ones, insufficient support in their work environment, and a lack of effective supportive treatments for their mental well-being (9, 15).

A recent meta-analysis revealed that during the COVID-19 outbreak, healthcare professionals experienced a pooled prevalence of 23.2% for anxiety, 22.8% for depression, and 38.9% for insomnia. Recognizing healthcare professionals as a valuable resource, it is crucial to prioritize their mental and physical well-being in both the short and long term to ensure their continued ability to cope with the ongoing and anticipated long-lasting impact of COVID-19 (12). International organizations such as the World Health Organization (WHO) have developed guidelines to enhance the mental well-being of healthcare professionals during the COVID-19 outbreak (12). The outbreak of COVID-19 has significantly strained Egypt’s already-overburdened healthcare sector, leading to rapidly overwhelming demand (16, 17). As is typical in disaster and conflict situations, healthcare workers may become targets of collective or political violence, as reported by the World Health Organization (18).

Several studies have assessed the rates of workplace violence in different healthcare settings in Egypt. Samir et al. reported that approximately 86% of nurses working in obstetrics and gynecology departments in Cairo had experienced workplace violence during the 6 months preceding their study in 2012 (19). The most common type of violence reported by female health workers was psychological, verbal abuse and disrespect, physical violence, and sexual harassment (20).

Furthermore, several studies instituted violence in similar settings in Egypt during the pandemic. At the emergency department in Ain Shams University Hospitals, 86.1% of the HCWs surveyed experienced verbal violence (14). A survey among Egyptian residents found verbal abuse was the most common, particularly from senior staff members (21). It is not only healthcare providers who are exposed to violence due to their work in healthcare facilities. A study conducted among security personnel working at a university hospital found that 87.3% of the respondents were exposed to violence within the previous year (22).

In Egypt, healthcare workers often work in an unprotected environment, with some departments without security guards. In departments where guards are present, they are often unarmed and ineffective due to several patient relatives accompanying the patient as part of the traditional Egyptian culture (19). This, coupled with a high level of illiteracy and low awareness about healthcare services and their complications, increases the likelihood of misunderstandings that can lead to violence (20).

Moreover, the presence of healthcare workers and patients in highly stressful environments makes them more susceptible to aggression (23). Interventions to prevent violence against healthcare workers in non-emergency settings focus on better managing violent patients and high-risk visitors. In contrast, interventions for emergency settings aim to ensure the physical security of healthcare facilities. Accordingly, further research is needed to assess the effectiveness of these programs, particularly in low-resource settings (6).

Considering the above, this study aims to understand the work environment of healthcare workers during the pandemic at Kasr Al-Aini University Hospital in Cairo, Egypt, and if they have received any training related to violence in healthcare settings. The study will assess the prevalence of violence against healthcare workers through a cross-sectional survey administered among healthcare workers.

## 2 Methods

A total of 183 Egyptian healthcare workers participated in a cross-sectional study through convenience sampling conducted over 6 months from November 2020 till the end of January 2021. They were recruited voluntarily and accessed an online self-administered questionnaire through social networks and emails, which they shared with their colleagues. The participants were not given incentives, and their responses were kept anonymous. After obtaining informed consent, participants completed the questionnaire. This article investigates the prevalence of workplace violence at health facilities in Egypt during the COVID-19 pandemic.

### 2.1 Study setting and population

A web-based survey about COVID-19 was distributed online through WhatsApp and Facebook platforms. The questionnaire was accessible via a hyperlink, and healthcare workers were the intended audience. This web-based electronic form was entirely voluntary and anonymous.

### 2.2 Sample size and technique

This study used a non-random convenience sample. The sample size was determined using Epi Info software, assuming a 90% power, 0.05 level of significance, and an estimated proportion of 82.5%, based on previous research (24). The required sample size will be 158 participants. To accommodate for potential dropout rates, an extra 10% was added. The final sample size targeted was calculated to be 174 participants.

### 2.3 Study tool and data collection technique

The study used an electronic questionnaire distributed through a link shared on social networks. The questionnaire included socio-demographic and occupational information, HCW knowledge and attitudes toward COVID-19, and HCWs’ exposure to violence during the COVID-19 pandemic. The questionnaire was modified from the WHO’s tool for behavioral insights on COVID-19 (25) and a study conducted in Turkey (26). The questionnaire was translated into Arabic and back-translated to English to ensure consistency with the original English version. A pilot testing phase was conducted to adapt the questionnaire to fit the Egyptian context and ensure clarity and comprehensibility of items and questions tailored to the culture of hospitals in Egypt. Approval was obtained from a panel of experts from the public health and community medicine department before submitting to the Research Ethics Committee, Faculty of Medicine Cairo University.

### 2.4 Data management

The collected data was coded and entered through Google Forms and Excel, then exported to SPSS version 21 (27). Descriptive statistics were used to summarize the data, including frequencies (n) and percentages (%) for qualitative variables and median and interquartile range (IQR) for non-normally distributed quantitative variables. Chi-square tests were used to analyze group comparisons for qualitative variables, with a significance level set at *p* ≤ 0.05.

## 3 Results

The survey was accessed by 200 respondents, of which only 183 consented and were included in these results, with a non-response rate of less than 10%. Overall, 42.5% of the respondents were females, and around 65% were above 30 years old. Respondents came from different departments; the highest was from the emergency department (35.5%) and 24.6% from the internal medicine department. The respondents span different essential functions in the hospital settings, where 47.5% were technicians and 21.3% were physicians in training. Furthermore, 73% of the respondents had more than 5 years of experience, and 33% did not receive any COVID-19-related training. The full breakdown of demographics can be found in Table 1.

**Table 1:** Demographic characteristics of the respondents.

	Frequency	Percentage
Gender		
Male	87	47.5
Female	96	52.5
Age in years		
20–30	63	34.4
31–40	81	44.3
41–50	36	19.7
51–60	3	1.6
Department		
-Many different hospital units/No specific unit	27	14.8
-Medicine (non-surgical)	45	24.6
-Surgery	6	3.3
-Pharmacy	3	1.6
-Laboratory	6	3.3
-Radiology	3	1.6
-Anesthesiology	10	5.5
-Emergency department	65	35.5
-Intensive care unit	18	9.8
Job title		
-Resident physician/physician in training	39	21.3
-Technician	87	47.5
-Registered nurse	15	8.2
-Physical therapist	6	3.3
-Head nurse	12	6.6
-Pharmacist	15	8.2
-Attending/staff physician	6	3.3
-Other	3	1.6
Years of experience		
Less than 1 year	12	6.6
1 to 5 years	39	21.3
6 to 10 years	54	29.5
11 to 15 years	60	32.8
16 to 20 years	18	9.8
Work modality		
Full-time. Part-time	126	68.9
On a contract	48	26.2
	9	4.9
Work shift		
Morning	51	27.9
Night	132	72.1
How many hours do you work per week in this unit? (12)		
<20 h/week	21	11.5
20–39 h/week	45	24.6
40–59 h/week	81	44.3
60–79 h/week	18	9.8
≥80 h/week	18	9.8
Have you received any COVID-19-related training courses?		
Yes	123	67.2
No	60	32.8

**Figure 1:**
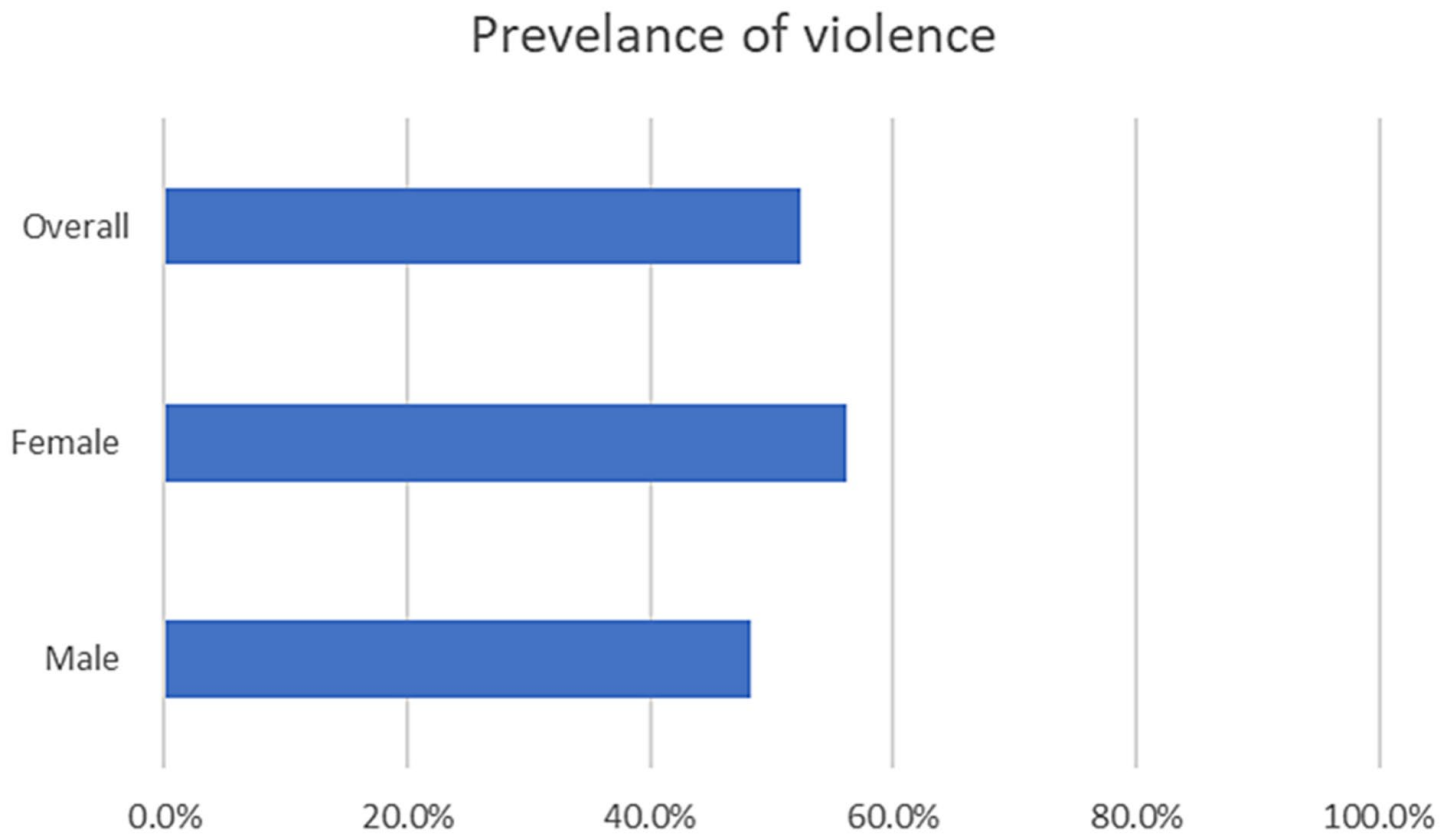
Frequency of violence amongst males and females.

After the demographic questions, the respondent was asked about COVID-19-related violence. Around 61% agreed that getting infected with COVID-19, and 88.5% of the respondents believed that their job in the healthcare sector was troublesome and a cause for problems. Furthermore, 59% stated that violence has increased during the pandemic, while 52.5% reported being subjected to violence. Of these, 97% stated that this violence was within the last 4 months of completing the questionnaire. Finally, 82% of the respondents did not receive any training on how to deal with violence in their line of duty. The violence prevalence by gender of the respondents is in Figure 1, where the breakdown for 96% of respondents were 54 females and 42 males.

Furthermore, the most common reaction to violence was reporting to their managers, putting barriers in dealing with others, and reporting to the police. Fear, disappointment, and anxiety were the most common feelings after an incident. The full breakdown is shown in Table 2.

**Table 2:** The violence trends during the pandemic and the respondents’ exposure.

Question	Frequency	Percentage
Do you think that COVID-19 infection is a stigma to those who become ill with it?		
Yes	111	60.7
No	72	39.3
Do you think your job leads to problems in the time of corona?		
Yes	162	88.5
No	21	11.5
In your opinion, what happened to the exposure to violence during the coronal disease outbreak?		
Decreased alot	63	34.4
Decreased	12	6.6
Increased	69	37.7
Increased a lot	39	21.3
Have you been subjected to any violence because of your career during the last 4 months?		
Yes	93	50.8
No	90	49.2
Have you been subjected to any violence during the COVID pandemic?		
Yes	96	52.5
No	87	47.5
Have you received any training on how to deal with violence in the line of duty before?		
Yes	33	18
No	150	82

Ninety-six respondents were subjected to violence and were asked about the type of violence, the perpetrators, and how they dealt with it. The most prevalent type was verbal violence; around 94% of the 96 respondents subjected to violence were exposed to verbal violence, followed by 44% for physical violence and 13% for sexual violence. The full breakdown can be found in Table 3. The most common perpetrator by far was the patients’ companions.

**Table 3:** The type of violence, the perpetrator, and how they deal with the violence, as per the respondents’ personal experience.

Question	Frequency	Percentage
Have you been subjected to physical violence in the previous 4 months? (96)		
Yes	42	43.75
No	54	56.25
Have you been subjected to verbal violence in the past 4 months? (96)		
Yes	90	93.75
No	6	6.25
Have you been verbally threatened in the past 4 months? (96)		
Yes	75	78.2
No	21	21.8
Have you been subjected to sexual harassment in the previous 4 months? (96)		
Yes	12	12.5
No	84	87.5
Who is the person who was violent toward you? (more than one answer is allowed) (96)		
-Patient	6	6.25
-Patient’s companion	78	81.25
-Fellow doctor	3	3.1
-Nurse	3	3.1
-Clerk	6	6.25
-Housekeeper	9	9.4
What do you currently do to deal with violence in your work? (more than one answer is allowed) (96)		
-I do nothing	3	3.1
-I put barriers in dealing with others	78	81.25
-I report to my manager at work	90	93.75
-I report to the police	42	43.75
-Show similar behavior	18	18.75
-I distance myself and leave the scene	6	6.25
-Pretend not to see the abuse	3	3.1
How do you feel after being subjected to violence? (more than one answer is allowed) (96)		
-Disappointment	39	40.6
-Sadness	21	21.8
-Powerlessness	18	18.75
-Low self-esteem	24	25
-Anger	27	28.1
-Hate	27	28.1
-Hostility/animosity	12	12.5
-Anxiety	39	40.6
-Despair	6	6.25
-Failure	18	18.75
-Shock/Astonishment	6	6.25
-Guilt or Shame	9	9.3
-Fear	45	46.8

**Figure 2:**
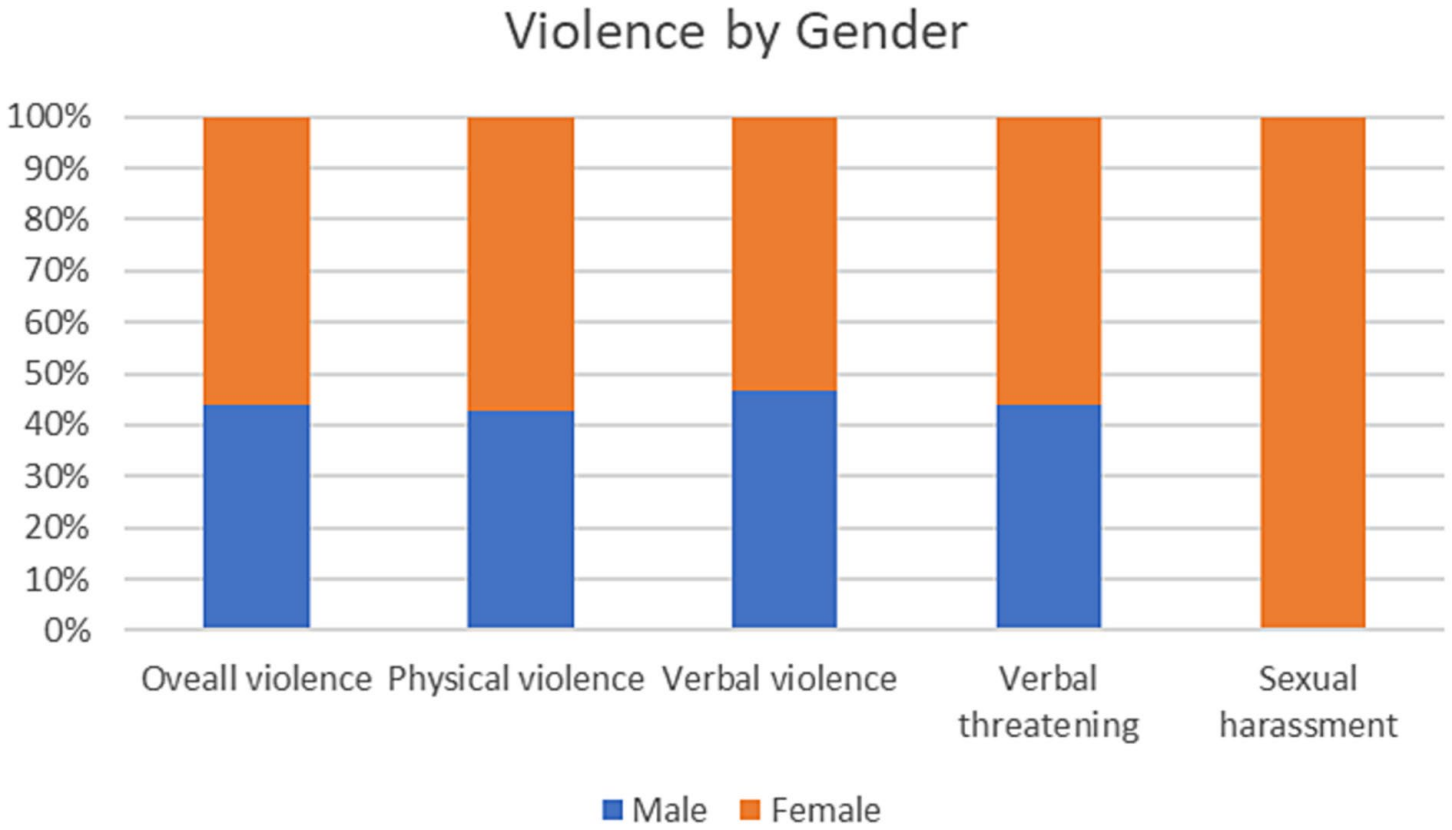
Type of violence by gender.

Figure 2 presents the prevalence of different types of violence by gender. The most common type of violence was verbal violence, declared by 90 respondents, of which 48 were females. This was followed by verbal threatening by 75 respondents, of which 42 are females, and then physical violence by 42, of which 18 are males. Sexual harassment was declared by 12 females only. The chi-square test did not show any significance by gender except for sexual harassment. The full results can be found in Annex 1.

Nevertheless, the type of violence showed a significant association with the age group, which was more prevalent amongst the 31–40 age group and showed a significant association with verbal violence, verbal threatening, and sexual harassment. The full breakdown with the *p*-values can be found in Annex 2. Additionally, the association between the perpetrator, response to violence, and the training received on how to deal with violence by gender was examined using the chi-squared test. The perpetrator and the response to violence questions revealed a significant association with a *p*-value <0.001, while the training received was non-significant with gender.

## 4 Discussion

This study showed that 52.5% of the respondents reported being subjected to violence during the pandemic. 93% reported verbal violence, 43% reported both verbal and physical, and 59% stated that violence increased during the pandemic. Those violence ratios are comparable to those reported at different Egyptian healthcare institutions during COVID-19, ranging from 10 to 40% for physical violence and an average of 80% for verbal violence (29, 30). However, those numbers are not different from those reported before the pandemic, according to the systematic review and meta-analysis by Stanely et al., who reported a ratio of 59.7–86.1 of healthcare workers in Egypt reporting violence in the workplace (31).

Numerous studies have shown that healthcare workers are more likely than people in other professions to become victims of violence. The troubling normalization of this issue worsens the increase in incidents and personal consequences for HCWs (24, 32). The situation worsened during the COVID-19 pandemic, dramatically influencing the mental health of all community sectors (33). Frontline HCWs were particularly vulnerable to those psychological effects because of their long working hours and inadequate personal protective equipment (PPE) (34, 35). Unfortunately, there were indications that violence toward HCWs rose during the COVID-19 pandemic, even though most communities worldwide understood their vital role during the pandemic (29, 36).

Female HCWs were the most frequent victims of violence (64.7%), consistent with a study in Iraq (37). Furthermore, the HCW who experienced any violence reported that patient companions were most likely to perform the violent act (81.2%), comparable to the results from similar studies in Egypt, India, and Ethiopia (38–40). This could be linked to the relatives of the patients being there on-site with patients. While the patients were being managed, their companions may have high levels of anxiety over the potential loss of the patients’ lives. Furthermore, their disregard for the visiting hours’ regulations and a substandard security system may have added to the problem. This is especially important when comparing such numbers to the United States, where a structured and safe work environment is ensured for all HCWs (41). Physical violence was the most commonly observed form, accounting for 84% of incidents. Among the targets of violence, nurses and unlicensed assistive personnel experienced it most frequently, making up 65% of the victims (42).

While disregarding the visiting hours, the low resources, and the substandard security system are essential in exacerbating the violence against healthcare workers; there is also a solid human factor to blame. Overall, 82% of our study respondents reported receiving no training for dealing with violent cases or adopting communication techniques to prevent them. A similar study was discussed in Saudi Arabia after legalizing laws for controlling workplace violence in healthcare settings. Although those measures decreased the prevalence of violence among healthcare workers, doctors still reported several incidents. The author contributed to the lack of knowledge and proper training in HCWs (43).

Only 18% of our respondents received any training on how to deal with violence in the workplace. A previous study by Abozaid et al. proposed and introduced training sessions for nurses on violence de-escalation. They assessed the impact of a training program for nurses in a teaching hospital in Egypt to enhance their capability to recognize and handle potentially violent circumstances effectively (44). This is an excellent approach for increasing the HCWs’ confidence in dealing with such incidents.

The current study has some limitations; firstly, it did not describe discrepancies between the public and private sectors, which will make all the difference as most private hospitals have good plans in action to avoid violence. Secondly, the issue of recall bias, as the survey questions were about past events. Furthermore, the study was based on convenient sampling. Thus, the reach was limited, challenging its generalizability as the study sample may not represent the entire population due to non-response, selection bias, or other factors. Additionally, since this is a cross-sectional study, this study’s type limitations should be considered. It cannot establish a cause-and-effect relationship between variables as it relies on self-reported data, which may be subject to recall or social desirability bias. Finally, cross-sectional studies do not provide information about the temporal sequence of events, making it challenging to determine whether exposure to a risk factor preceded or vice versa.

To address the limitations of the present study, further investigation is necessary. The following recommendations are proposed: Firstly, it is imperative to implement an effective management and reporting system in healthcare institutions, with a focus on taking swift action and monitoring occurrences. Secondly, conducting multiple public awareness campaigns to enhance knowledge about the detrimental impacts of workplace violence on patients and healthcare workers while also improving overall healthcare quality. Finally, healthcare professionals should receive training on formal reporting mechanisms, and such educational activities should be integrated into the curricula of medical schools and residency programs.

In conclusion, this research revealed the prevalence of violent assaults in healthcare settings, predominantly through verbal attacks perpetrated by patients’ families or acquaintances. This issue is of great concern, as it has been observed that despite reporting such incidents, most respondents did not witness any significant action taken by the government. The study also highlighted the importance of training healthcare professionals in dealing with workplace violence. Moreover, the study found that the COVID-19 pandemic did not significantly alter the frequency of violent incidents reported by healthcare workers, suggesting that the underlying causes of violence are systemic and extend beyond the pandemic era.

## 5 Informed consent statement

“Your participation in this online survey is completely voluntary. All of the information that you provide for the study will be kept completely confidential. We only record your responses, but the questionnaire will not have your name on it, and your responses to our questions are identified only by a number, never by name. We hope that this survey will be considered as a baseline assessment that will guide us in improving the work environment of the HCPs which is considered an important step in combating the infection. The survey will take about 10–15 min.”

## Data availability statement

The original contributions presented in the study are included in the article/Supplementary material, further inquiries can be directed to the corresponding author.

## Ethics statement

This study was approved by the Research Ethical Committee at the Faculty of Medicine, Cairo University (Approval No. 110-2020). The study was conducted in accordance with the Helsinki Declaration (22). Informed consent was obtained from all respondents before completing the survey voluntarily. A statement for informed consent was included in the online survey before the start of the survey. Respondants giving their voluntary informed consent were directed to the survey questions. The studies were conducted in accordance with the local legislation and institutional requirements. The participants provided their written informed consent to participate in this study.

## Author contributions

SS: Formal analysis, Methodology, Writing – original draft. RA: Supervision, Validation, Writing – review & editing. AA: Conceptualization, Resources, Writing – original draft. AN: Investigation, Resources, Writing – original draft. RE: Investigation, Methodology, Software, Supervision, Writing – review & editing. AH: Conceptualization, Resources, Validation, Writing – original draft.
