# Supplementary material for: Violence against healthcare workers during the COVID-19 pandemic: a cross-sectional survey at Cairo University Hospital
Source: Front Public Health. 2023 Nov 16;11:1277056. doi: 10.3389/fpubh.2023.1277056 (PMC10693415; doi:10.3389/fpubh.2023.1277056)
Supplement: Supplementary file 1 [file Table_1.docx]

# Annex 1

Annex 1 relation between socio-demographics and violence:

|  | Gender | | p-value* | Age | | | | p-value* |
| --- | --- | --- | --- | --- | --- | --- | --- | --- |
|  | male | female |  | 20-30 | 31-40 | 41-50 | 51-60 |  |
| Subjected to any kind of violence | 42  43.75% | 54  56.25% | 0.281 | 30  31.3% | 51  53.1% | 6  6.3% | 9  9.4% | 0.378 |
| Physical violence | 18  42.9% | 24  57.1% | 0.489 | 18  42.9% | 18  42.9% | 0  0% | 6  14.3% | **0.008** |
| Verbal violence | 42  46.7% | 48  53.3% | 0.816 | 27  30% | 45  50% | 9  10% | 9  10% | **0.005** |
| Verbal threatening | 33  44% | 42  56% | 0.424 | 18  24% | 42  56% | 9  12% | 6  8% | **0.003** |
| Sexual harassment | 0  0% | 12  100% | **0.001** | 0  0% | 6  50% | 0  0% | 6  50% | **<0.001** |

# Annex 2

Annex 2 Other covid-19 questions not directly related to violence in the workplace

| **Have you received any COVID-19-related training courses?**  Yes  No | 123  60 | 67.2  32.8 |
| --- | --- | --- |
| **Have you received any COVID-19-related infection control cycle?**  Yes  No | 99  84 | 54.1  45.9 |
| **Does your hospital provide a protocol for the treatment of COVID-19?**  Yes  No | 150  33 | 82.0  18.0 |
| **Are all IPC and PPEs readily available at your healthcare facility?**  Yes  No | 105  78 | 57.4  42.6 |
| **Do you feel well prepared to deal with any possible corona sitCOVID-19 case?**  Yes  No | 75  108 | 41.0  59.0 |
| **In your opinion, how dangerous is the new Coronavirus?**  Extremely Dangerous  More or less dangerous  Not Dangerous | 19  86  78 | 10.4  47.0  42.6 |
| **Who do you think is most at risk of contracting COVID-19 infection?**  Youth  Adults  Seniors  Pregnant Ladies  Health care workers | 12  12  57  3  99 | 6.6  6.6  31.1  1.6  54.1 |
| **Do you think you're likely to be infected with COVID-19?**  Yes  No  I Don't Know | 126  24  33 | 68.9  13.1  18.0 |
| **What have you and your family done to prevent COVID-19 infection in recent days? (more than one option allowed)**  -Wash hands regularly using an alcohol-based hand wash or soap and water.  - Cover the mouth and nose when coughing or sneezing.  -Avoid close contact with anyone with fever and cough.  -Get rid of stagnant water.  -Cook meat and eggs well  -Avoid direct unprotected contact with live animals and animal contact surfaces. | 27    108  87  25  6  9 | 14.8    59  47.5  13.66  3.2  4.9 |
| **Do you think it is important to take action to prevent the spread of COVID-19 within your community?**  Yes  No | 159  24 | 86.9  13.1 |
| **What would you do if you or your family member was infected with the new coronavirus? (more than one option allowed)**  -I'll go to the hospital or to the health unit.  -I'm going to buy medicines from the pharmacy.  - I'll stay in the house quarantine. | 63  42  90 | 34.4  22.95  49.2 |
| **Have you had any of these symptoms lately?**  -**Had** **no symptoms**  - Fever.  - Cough.  - Shortness of breath and difficult breathing.  - Muscle pain.  - Headache.  - Diarrhea. | **63**  84  45  39  42  27  36 | **34.4**  45.9  24.6  21.3  22.9  14.8  19.6 |
| **Have you received COVID-19 PCR analysis?**  Yes  No | 78  105 | 42.6  57.4 |
| **If answer is yes, Was the result of the PCR positive?** **(78)**  Yes  No | 63  15 | 80.8  19.2 |
| **If answer is yes, What was your case severity? (63)**  Mild  Moderate  Severe | 6  36  21 | 9.53  57.14  33.33 |
| **If answer is yes, As a corona patient, have you been subjected to any kind of violence? (63)**  Yes  No | 9  54 | 14.3  85.7 |
